# Supplementary material for: High-speed energy-efficient memristor confined in sub-5 nm space with elemental oxygen reservoir layer
Source: Nat Commun. 2026 Mar 18;17:4117. doi: 10.1038/s41467-026-70806-4 (PMC13149778; doi:10.1038/s41467-026-70806-4)
Supplement: Supplementary file 1 — Supplementary Information [file 41467_2026_70806_MOESM1_ESM.pdf]

Supplementary Information

High-speed energy-efficient memristor confined in sub-5 nm space with elemental oxygen reservoir layer

Chenfei Li[1,#], Wencheng Niu[2,#], Da Wan[1*], Lin Tang[2], Zhengdao Xie[2], Kai Zhang[1], Yuan Liu[2], Qi Liu[3], Lei Liao[2*], Xuming Zou[2*], Xingqiang Liu[2*]

[1]School of Electronic Information, Wuhan University of Science and Technology, Wuhan 430081, China.

[2]State Key Laboratory for Chemo/Biosensing and Chemometrics, College of Semiconductors (College of Integrated Circuits), Hunan University, Changsha 410082, China.

[3]The Frontier Institute of Chip and System, Fudan University, Shanghai 200433, China.

[#]These authors contributed equally: Chenfei Li, Wencheng Niu.

*Corresponding authors. Da Wan, wanda@wust.edu.cn; Lei Liao, liaolei@whu.edu.cn; Xuming Zou, zouxuming@hnu.edu.cn; Xingqiang Liu, liuxq@hnu.edu.cn

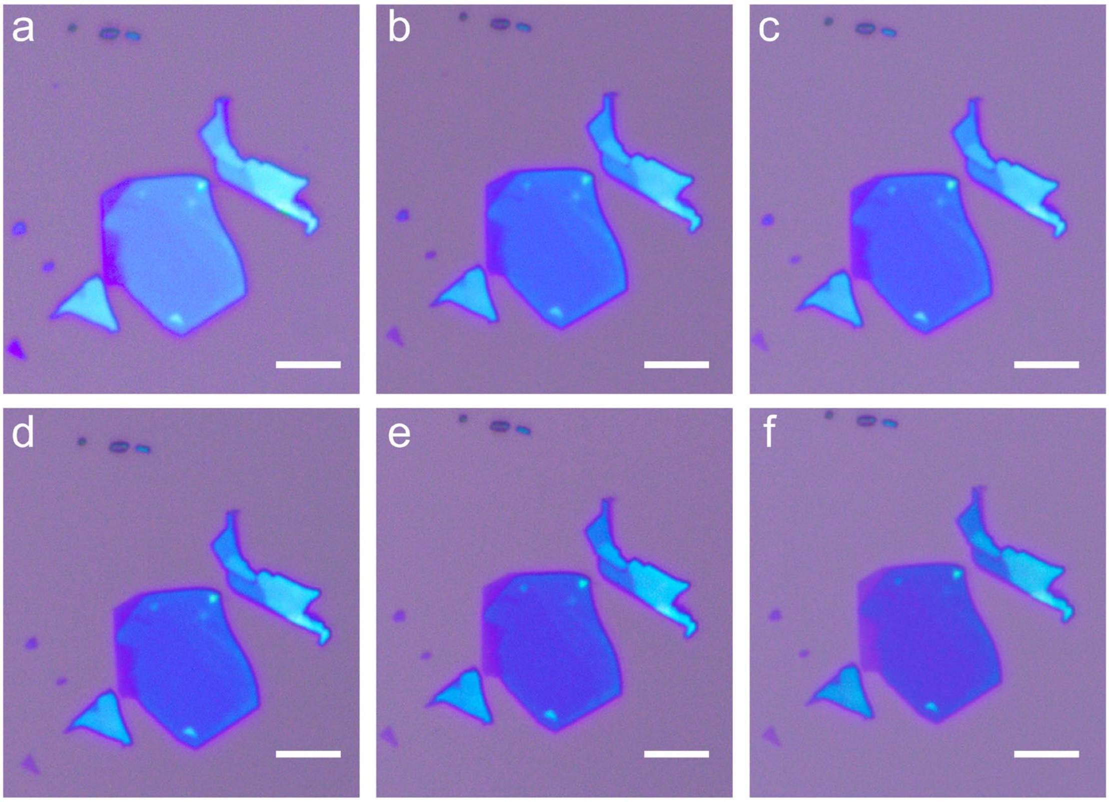

Supplementary Fig. 1 | Optical images of HfS_2 flakes under different ozone treatment time. a 5 min. **b** 10 min. **c** 20 min. **d** 30 min. **e** 40 min. **f** 60 min. Scale bar: 5 μm.

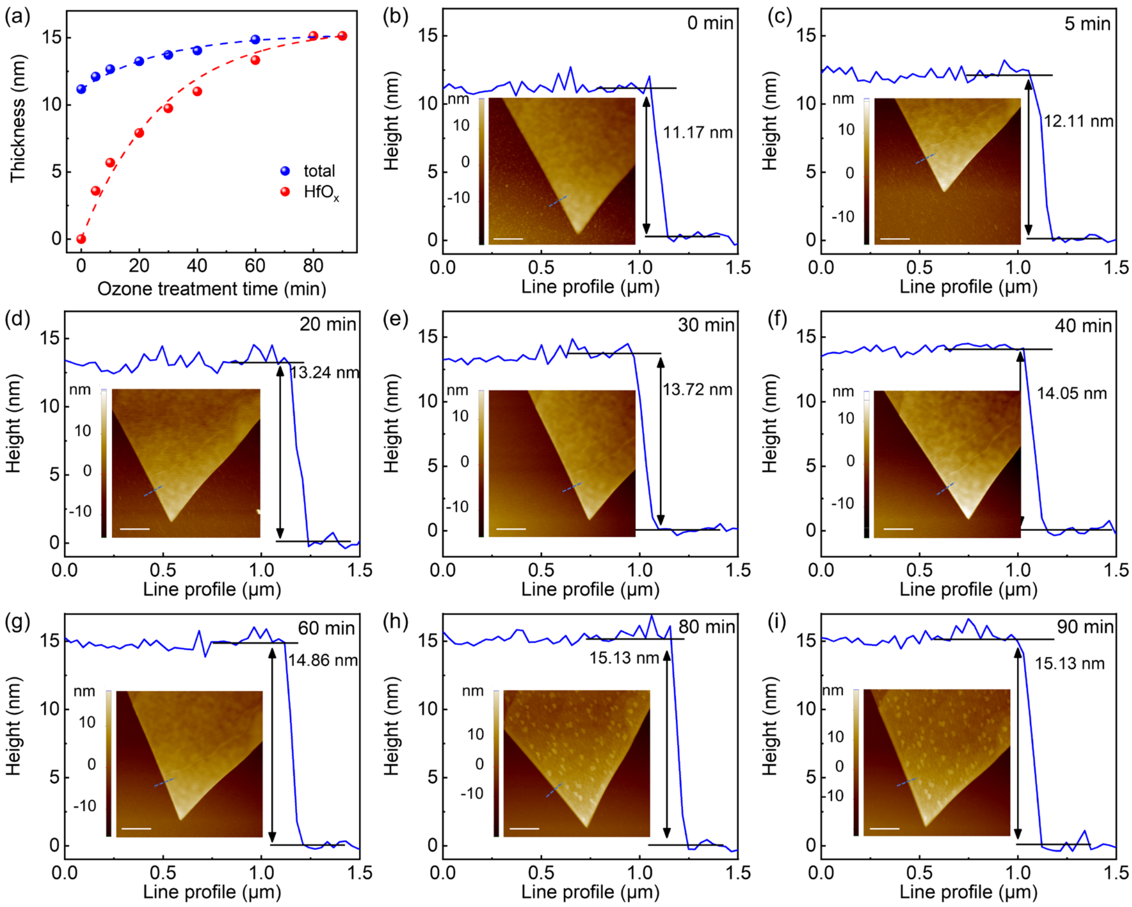

Supplementary Fig. 2 | AFM images and thickness evolution during the transformation of HfS_2 into HfO_x. a Total thickness of the $HfO_x/HfS_2/HfO_x$ stack and the HfO_x thickness as a function of ozone treatment time. **b-i** Evolution of height profiles with varying ozone treatment time. Each inset shows an AFM image. Scale bar: 2 μm. With increasing ozone treatment time, the HfS_2 flake gradually transforms from a partially oxidized state to a completely converted HfO_x layer. Based on the initial thickness of HfS_2 (11.17 nm, Fig. 2b) and the final thickness of the fully oxidized sample (15.13 nm, Fig. 2i) measured by atomic force microscopy, the expansion ratio was determined to be 1.35. To quantify the thickness of the converted HfO_x at various stages, we defined two parameters: the total AFM-measured thickness (t) and the thickness of the residual unconverted HfS_2 (x). Their relationship is expressed by the following equation: $t=x+1.35\times(11.17-x)$. By determining the value of x, the thickness of the formed HfO_x layer can be precisely derived.

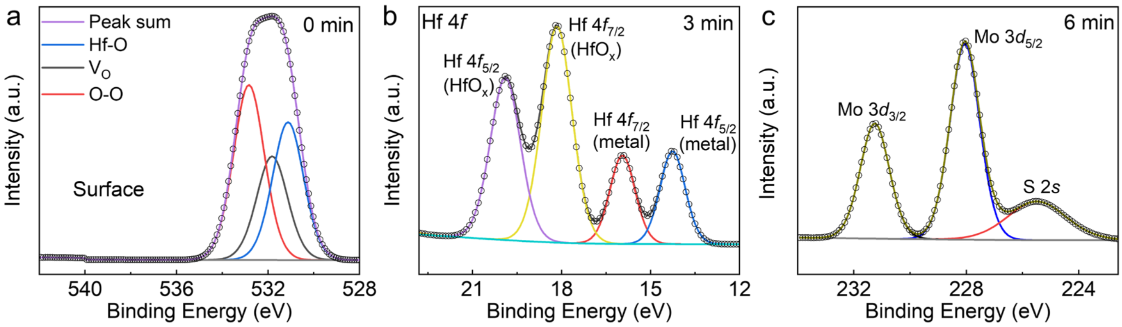

Supplementary Fig. 3 | Depth-resolved XPS spectra. **a** O 1*s* spectra collected at the surface. **b** Hf 4*f* spectrum obtained after 3 min of etching. **c** Mo 3*d* spectrum acquired after 6 min of etching.

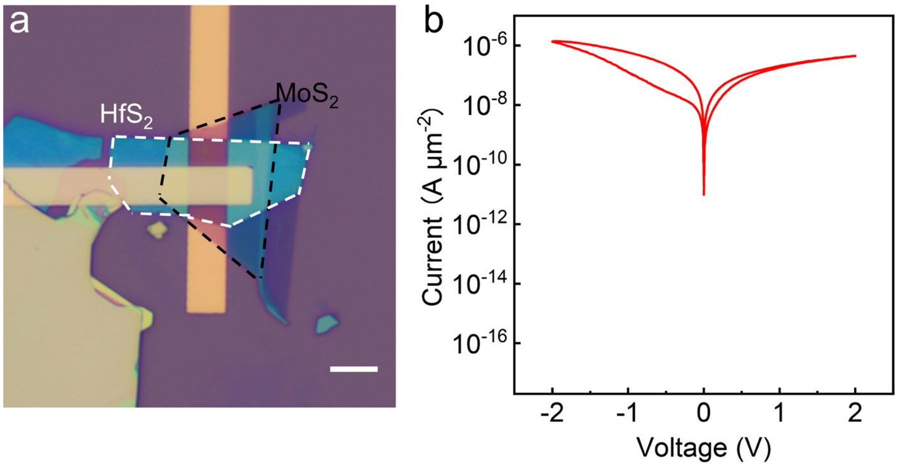

Supplementary Fig. 4 | Electrical characteristics of the control MoS_2/HfS_2 device. **a** Optical microscopy image of the device without ozone treatment. Scale bar: 5 μm. **b** I-V curves exhibiting negligible resistive switching behavior.

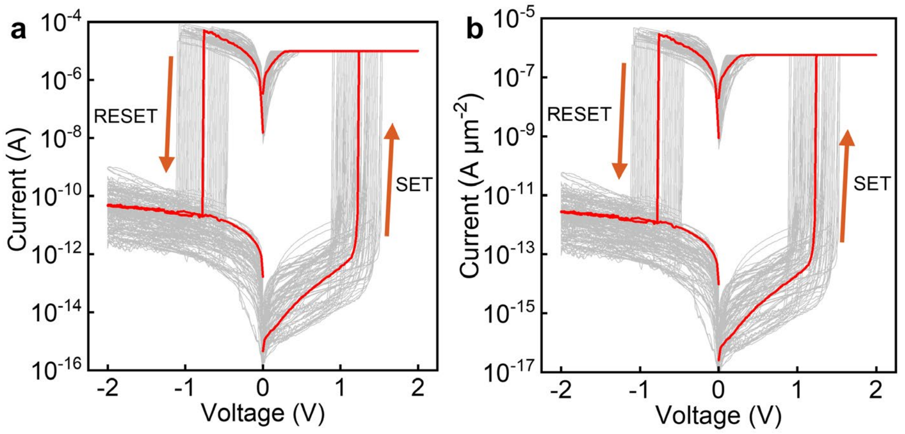

Supplementary Fig. 5 | Electrical performance of the EOR-based memristor array. **a** Typical I-V characteristics plotted on an absolute current scale. **b** Corresponding I-V curves normalized by device area, shown as current density.

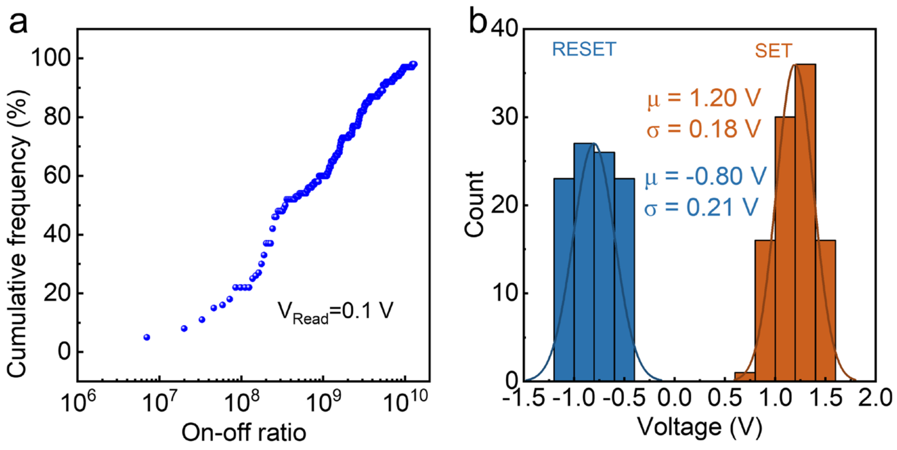

Supplementary Fig. 6 | a Cumulative probability distribution of the on/off current ratio extracted from I-V measurements of 100 memristors (at V_{READ} = 0.1 V). **b** Statistical distribution of SET and RESET voltages across 100 devices.

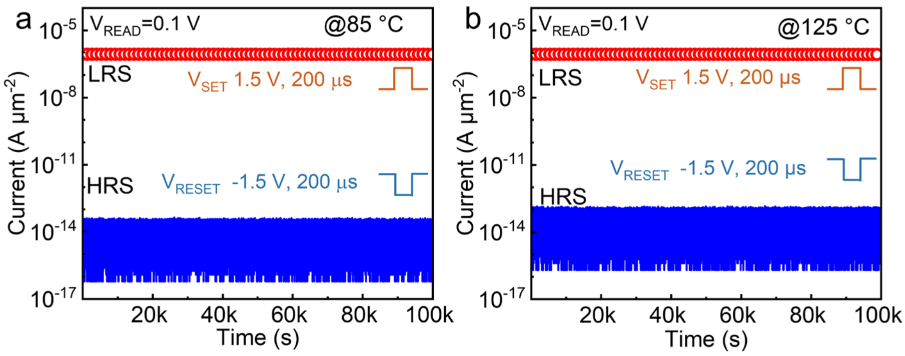

Supplementary Fig. 7 | Retention characteristics. Measured at (a) 85 °C and (b) 125 °C, respectively.

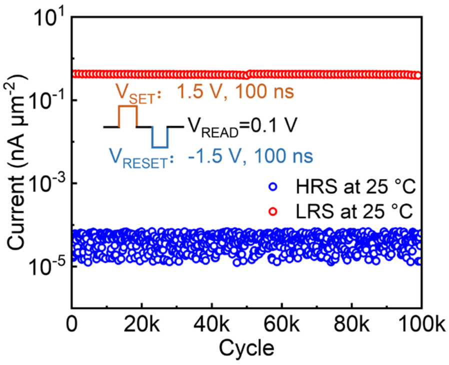

Supplementary Fig. 8 | Endurance characteristics. The device was monitored over 10^5 cycles using bipolar voltage pulses of ±1.5 V (pulse width: 100 ns).

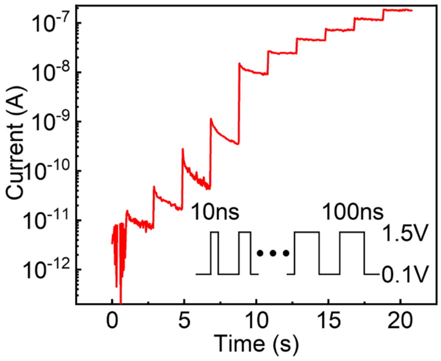

Supplementary Fig. 9 | Read current of the EOR-based memristor measured at different pulse widths.

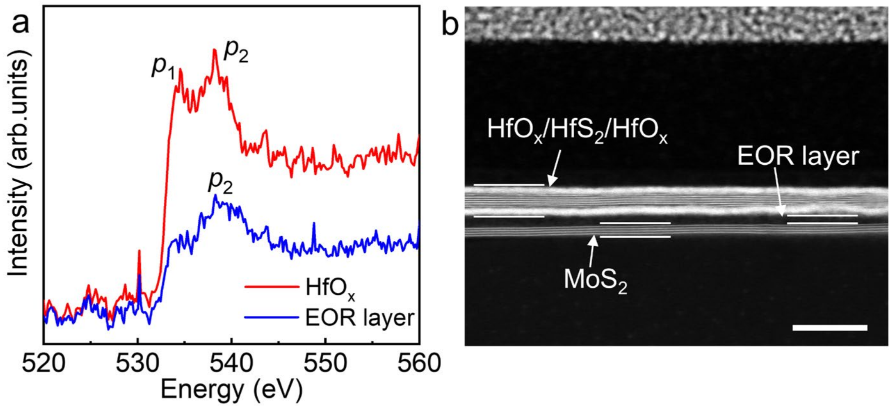

Supplementary Fig. 10 | ELNES spectra and TEM analysis. a O K-edge ELNES spectra acquired from the HfO_x switching layer and the EOR layer, respectively. **b** Cross-sectional cross profile of the TEM sample. Scale bar: 20 nm.

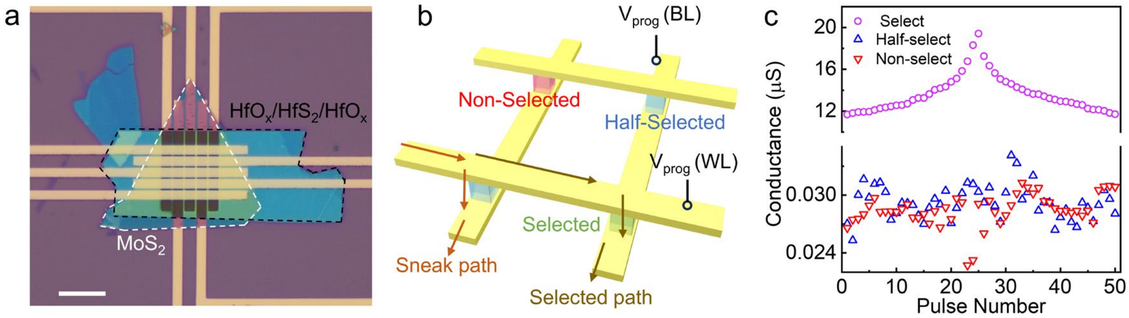

Supplementary Fig. 11 | Sneak-path analysis of the memristor arrays. a Optical image of a 5 × 5 memristor array. Scale bar: 20 μm. **b** Schematic illustration showing the definitions of different device states within the array. V_{prog} denotes the programming voltage applied to the word lines (WLs) to induce resistive switching in the selected cell. **c** Conductance evolution of selected, half-selected, and unselected cells in the array during pulse programming (±1.5 V, 20 ns).

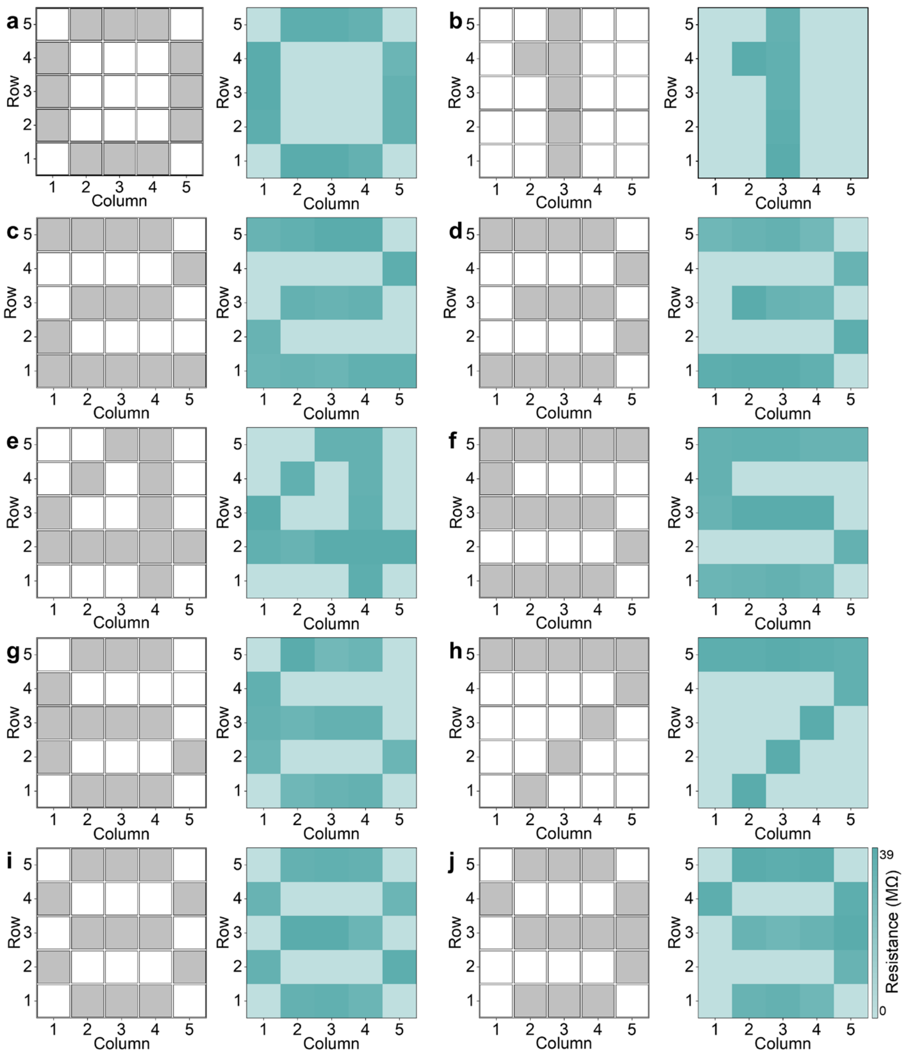

Supplementary Fig. 12 | Demonstration of digital pattern storage in a 5 × 5 crossbar memory array. a-j Schematic diagrams (left) and corresponding experimental heatmaps (right) of the programmed patterns for digits “0” through “9”, respectively. For the schematic diagrams, the gray squares represent the high-resistance state (HRS), while the white squares represent the low-resistance state (LRS). The experimental heatmaps show the measured resistance values of each memory cell after pulse programming, where the color scale (z-axis) indicates the resistance level. The x and y axes denote the device coordinates (column and row indices). All patterns were successfully written into and read from the array, demonstrating the reliability of the device for digital data storage.

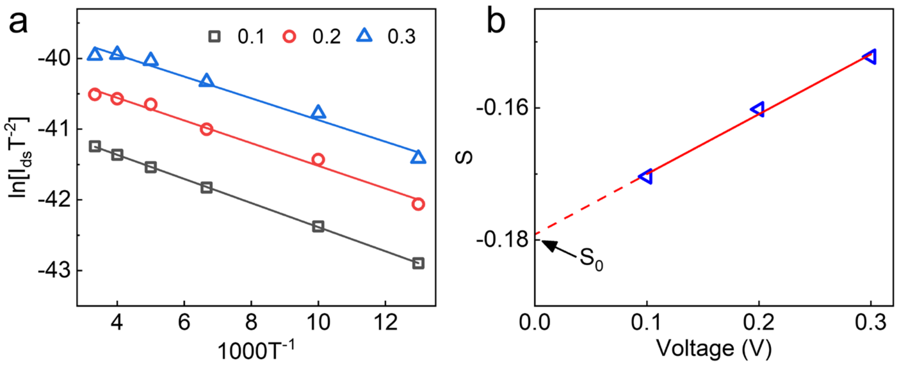

Supplementary Fig. 13 | a Arrhenius plot showing the dependence of IT^{-2} on inverse temperature T^{-1}, where the solid lines represent linear fits. **b** Dependence of the extracted slope *S* on the bias voltage. Extrapolation to zero bias yields S_0, which is utilized to calculate the effective Schottky barrier height.

Supplementary Table. 1 | Comparison of on/off ratio, normalized HRS current, switching time, and energy consumption with various advanced synaptic devices.

Material	On/off ratio	HRS current ($A\ \mu m^{-2}$)	Switching time (ns)	Energy consumption (fJ)	Refs.
HfO_x/MoS_2	1.5×10^9	1.5×10^{-15}	8	0.093 fJ	This work
MoS_2	10^7	10^{-13}	600	4.5 fJ	[17]
2H-MoS_2	10^3	10^{-8}	17	700 fJ	[24]
$MoS_{2-x}O_x$	10^2	10^{-4}	100	-	[10]
WS_2	10^3	10^{-10}	13	299.8 fJ	[25]
BP	3.6×10^7	10^{-11}	40	20 pJ	[26]
hBN	10^2	10^{-10}	0.12	2 pJ	[27]
MoS_2/hBN	10^6	10^{-12}	20	3 pJ	[28]
In_2Se_3/h-BN	10^9	4.2×10^{-13}	-	-	[29]
IPS	4×10^7	10^{-11}	20	0.056 fJ	[30]
$HfSe_xO_y$	10^3	6×10^{-12}	50	0.1 fJ	[31]
SnS	10^8	10^{-12}	1.5	100 fJ	[32]
Bi_2O_2Se	10^3	10^{-9}	5	3.02 pJ	[33]
